# Novel Dual-band Band-Pass Filters Based on Surface Plasmon Polariton-like Propagation Induced by Structural Dispersion of Substrate Integrated Waveguide

**DOI:** 10.1038/s41598-018-26705-w

**Published:** 2018-05-29

**Authors:** Norbert Cselyuszka, Zarko Sakotic, Goran Kitic, Vesna Crnojevic-Bengin, Nikolina Jankovic

**Affiliations:** BioSense Institute—Research Institute for Information Technologies in Biosystems, University of Novi Sad, Dr Zorana Djindjica 1a, 21101 Novi Sad, Serbia

## Abstract

In this paper, we present two novel dual-band bandpass filters based on surface plasmon polariton-like (SPP-like) propagation induced by structural dispersion of substrate integrated waveguide (SIW). Both filters are realized as a three-layer SIW where each layer represents a *sub-SIW* structure with intrinsic effective permittivity that depends on its width and filling dielectric material. The layers are designed to have effective permittivities of opposite signs in certain frequency ranges, which enables SPP-like propagation to occur at their interfaces. Since three layers can provide two distinct SPP-like propagations, the filters exhibit dual-band behaviour. A detailed theoretical and numerical analysis and numerical optimization have been used to design the filters, which were afterwards fabricated using standard printed circuit board technology. The independent choice of geometrical parameters of *sub-SIWs* and/or the corresponding dielectric materials provide a great freedom to arbitrarily position the passbands in the spectrum, which is a significant advantage of the proposed filters. At the same time, they meet the requirements for low-cost low-profile configuration since they are realized as SIW structures, as well as for excellent in-band characteristics and selectivity which is confirmed by the measurement results.

## Introduction

Surface plasmon polaritons (SPP) present electromagnetic waves that occur at the interface of a dielectric and conductor due to the coupling of light to collective electron oscillations^1^. Owing to the specific nature, SPPs allow for breaking the diffraction limit and the localization of light into subwavelength dimensions, ultimately enabling strong field enhancements. As such, SPPs have been applied in various fields including optical communications, photonics, and sensing^2–5^.

The advantageous properties of SPPs such as slow-wave behaviour and field confinement are also very desirable in frequency regimes other than optical since they open up possibilities for significant improvement of components and devices. Since SPPs naturally occur at optical frequencies, there have been proposed different concepts to engineer SPP phenomenon in other frequency ranges including microwaves and terahertz.

One of the concepts that effectively mimics SPPs are spoof or designer surface plasmon polaritons^6,7^, which have been widely used in different microwave circuits including transmission lines^8–16^, filters^17–26^, antennas^27^, couplers^28^, splitters^29^, absorbers^30^, and circulators^31^. Unlike genuine SPPs, spoof SPPs are supported by specifically designed structures, predominantly by grooved strips whose geometrical properties are used to tailor the behaviour of spoof SPPs.

Recently, a novel “natural” SPP-like concept in microwave regime has been proposed, which is based on exploitation of the well-known structural dispersion of the electromagnetic modes in parallel-plate waveguide structure filled only with materials with positive permittivity^32,33^. Namely, if a parallel-plate waveguide is divided into two parts by an array of wires placed along the plane normal to the plates, and if the parts are filled with different materials, then the two parts exhibit effective permittivities of opposite signs in a certain frequency range, which allows for SPP to occur at their interface. This methodology was applied in somewhat different waveguide structure - substrate integrated waveguide (SIW), and the realized configuration demonstrated potential for filtering applications at microwave frequencies^34^.

It is well-known that the rapid growth of communication systems imposes the requirements for high-performance, low-cost, low-profile components that operate at two or more non-harmonically related frequencies. Taking into consideration those demands and the potential of the proposed concept, in this paper we propose two novel dual-band filters that employ “natural” SPP-like phenomenon, to achieve operation at two non-harmonically related microwave frequencies. Besides slow-wave behaviour and field confinement, SPP-like propagation also provides a transmission zero in spectral response, capable to clearly separate a passband in the spectrum, which is the underlying idea of filtering operation based on SPP-like propagation.

The proposed filters are realized as a microstrip-fed three-layer SIW where each layer represents a *sub-SIW* structure with intrinsic structural dispersion depending on its geometry and dielectric material. One filter comprises *sub-SIW* structures of the same width but with different dielectric materials, whilst the other one is comprised of three *sub-SIWs* which are filled with the same dielectric material, but have different widths. Since the layers are designed to have effective permittivities of opposite signs in certain frequency ranges, two distinct SPP-like propagations occur at the interfaces between the top and middle *sub-SIWs* and the middle and bottom *sub-SIWs*, which ultimately provides two passbands in the filters’ response.

To the best of authors’ knowledge, the two filters are first dual-band microwave filters based on “natural” SPP-like concept. The independent choice of geometrical parameters of *sub-SIWs* and/or the corresponding dielectric materials provide a great freedom to arbitrarily position the passbands in the spectrum, which is a significant advantage of the proposed configurations. At the same time, they meet the requirements for excellent in-band characteristics and selectivity as well as the requirements for low-cost low-profile configuration since they are realized as SIW structures.

In the following sections, a detailed theoretical background of the filters’ design will be presented, followed by the numerical analysis and discussion on their performance. The fabrication process will be described, and the measurement results of the fabricated circuits will be presented to confirm the high potential of the proposed filters.

## Results

### Theoretical analysis

**Figure 1:**
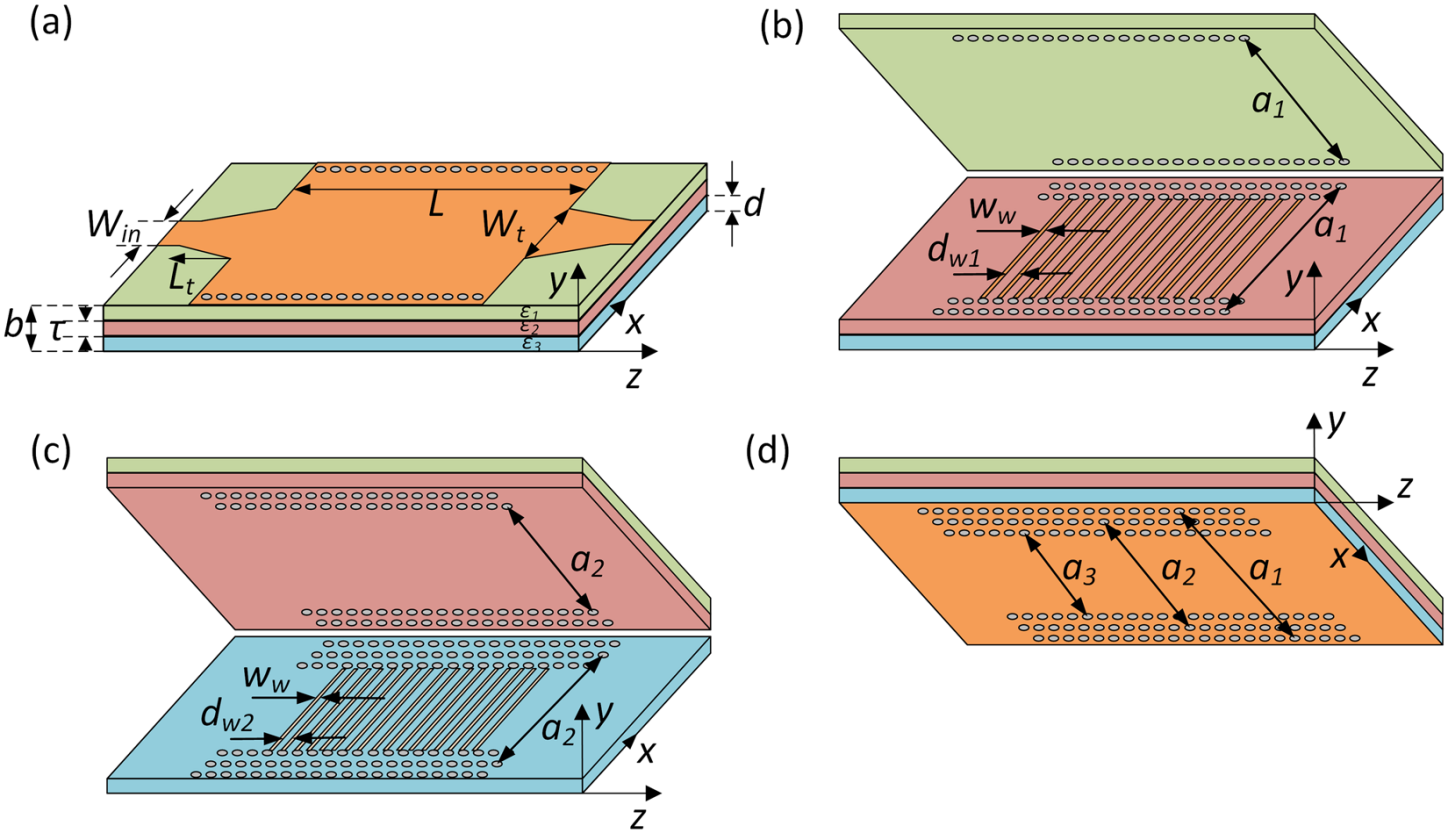
The layout of the proposed filters. (**a**) Overall structure. (**b**) Interface between the top and the middle *sub-SIWs*. (**c**) Interface between the bottom and the middle *sub-SIWs*. (**d**) Bottom side of the bottom *sub-SIW*.

The layout of the proposed filters is shown in Fig. 1 together with the corresponding geometrical parameters. The filters are based on a substrate integrated waveguide that is fed by tapered microstrip lines. Being bounded by metal walls on the top and bottom sides, and by arrays of vias on lateral sides, SIW represents a waveguide structure whose dominant propagating mode is TE_10_, whilst TM modes are not supported. The effective dielectric constant of TE_10_ mode can be defined as^35^:

1 $${\varepsilon }_{e}={\varepsilon }_{r}-{(\frac{c}{2af})}^{2}$$

where *f* is frequency, *c* is the speed of light, *a* is the width, and *ε*_*r*_ dielectric constant of the material that fills the SIW. The cut-off frequency is defined as

2 $${f}_{cTE10}=\frac{c}{2a\sqrt{{\varepsilon }_{r}}},$$

and below it the effective dielectric constant is negative, i.e. the wave propagation is not allowed, whilst above it the propagation is allowed. Such structural dispersion of SIW indicates that SPP-like propagation can be achieved by coupling of two TE_10_ modes, providing the modes have effective dielectric constants of opposite signs. In other words, SPP-like propagation can be achieved at the interface of two SIWs in the frequency range in which the two have effective dielectric constants of opposite signs, and this is the underlying idea of the proposed filters.

To achieve SPP-like propagation in the proposed configuration, the SIW is divided into three layers, i.e. there are three *sub-SIWs* in the proposed structure, Fig. 1. In general case, the three *sub-SIWs* have different widths *a*_1_, *a*_2_, and *a*_3_ and they are filled with different dielectric materials whose dielectric constants are *ε*_1_, *ε*_*2*,_ and *ε*_3_, respectively. Since effective dielectric constant of a *sub-SIW* depends on its width and the filling dielectric material, by the proper choice of those parameters we can tailor the frequency ranges in which the three *sub-SIWs* have desirable sign of the effective dielectric constant.

**Figure 2:**
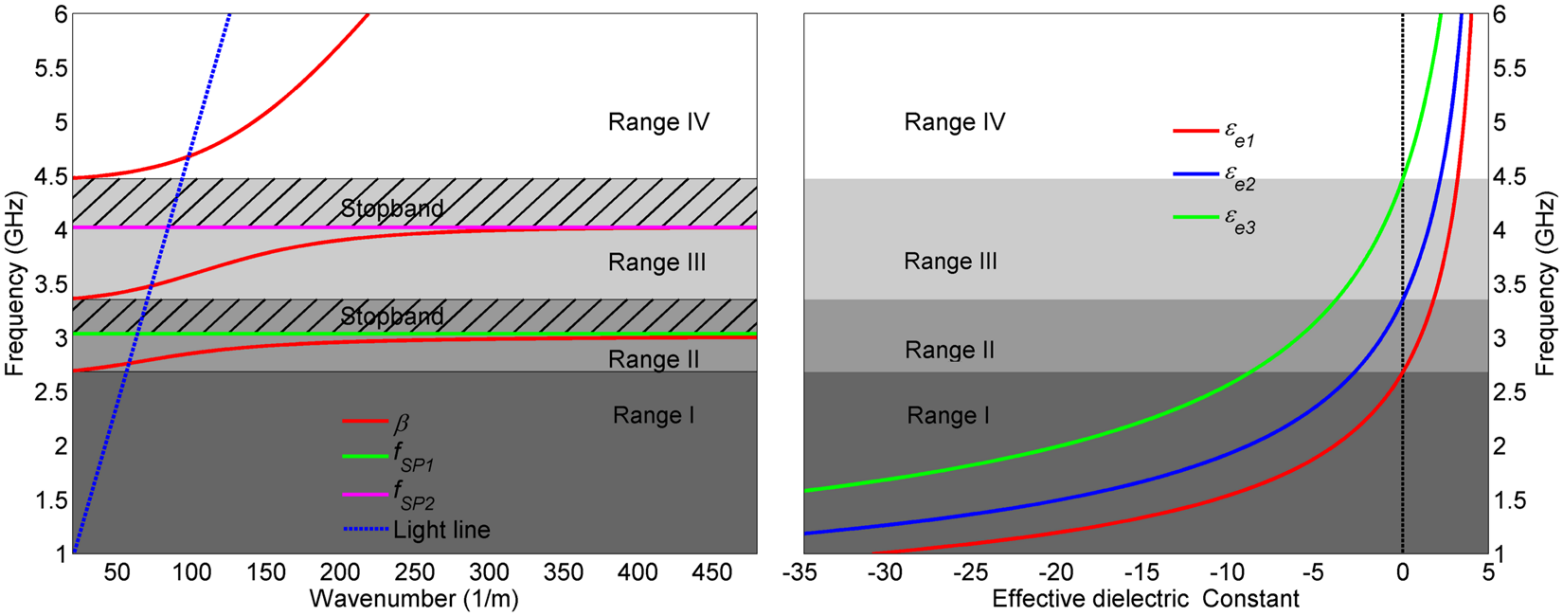
Frequency ranges according to the signs of the effective dielectric constants of TE_10_ mode in the three *sub-SIWs* and the corresponding dispersion curve. The geometrical parameters of the structure are the following: *a*_*1*_ = 25 mm, *a*_2_ = 20 mm, *a*_3_ = 15 mm, *ε*_1_ = *ε*_2_ = *ε*_3_ = 5, *d* = *τ* = 1 mm and *b* = 3 mm.

If we consider that the top *sub-SIW* has the lowest, and the bottom *sub-SIW* the highest cut-off frequency, then Fig. 2 summarizes the four frequency ranges according to the signs of the effective dielectric constants of TE_10_ mode in the three *sub-SIWs*. Whilst the first frequency range entirely forbids propagation, and the fourth allows conventional propagation typical for a SIW, the second and the third ranges are of a particular interest for the filters’ design. Namely, in the second range the top and the middle *sub-SIWs* exhibit effective dielectric constants of opposite signs, thus allowing for SPP-like propagation at their interface. By the same token, in the third frequency range SPP-like propagation occur at the interface between the middle and the bottom *sub-SIWs*.

Since the proposed structure can support SPP-like propagation in two different frequency ranges, this opens up possibility to design dual-band filtering operation. To further explain how the passbands are actually formed, the dispersion relation of the structure should be considered. Although not partially filled waveguide *per se*, for the sake of theoretical analysis the whole SIW can be considered in that manner. The wavenumber in the three *sub-SIWs* can be defined as^36,37^:

3 $${k}_{yi}=\sqrt{{\beta }^{2}-{k}_{0}^{2}{\varepsilon }_{ei}}$$

where *β* is the propagation constant in the *z* direction, *k*_0_ is the wavenumber in vacuum, *k*_*yi*_ is the wavenumber in the *y* direction, *ε*_*ei*_ effective dielectric constant, whilst *i* take the values 1, 2, and 3, denoting the top, middle, and bottom layer, respectively. Without loss of generality, the losses are omitted to simplify the explanation.

The dispersion relation for the overall structure can be obtained by solving the wave equation and imposing boundary conditions:

4 $$\begin{array}{c}\frac{{k}_{y3}}{{\varepsilon }_{e3}}tanh({k}_{y3}d)=-\frac{{k}_{y2}}{{\varepsilon }_{e2}}\,{\coth }({k}_{y2}\frac{\tau }{2}+\psi ),\\ -\frac{{k}_{y1}}{{\varepsilon }_{e1}}tanh({k}_{y1}(b-d-\tau ))=\frac{{k}_{y2}}{{\varepsilon }_{e2}}\,{\coth }({k}_{y2}\frac{\tau }{2}-\psi ),\end{array}$$

where *d*, *τ*, and *b* are the geometrical parameters of the structure as shown in Fig. 1(a), and *k*_*iy*_ are the wave-vector components along *y* direction in each layer. A detailed derivation of the Eq. (4) and explanation of the parameter *Ψ* are given in the Supplementary Material.

To illustrate the behaviour of the propagation constant and the overall SIW, Fig. 2 also shows the dispersion curve of the proposed structure. One can distinguish the four frequency ranges that were previously discussed – in the first range all *sub-SIWs* have negative effective permittivity and thus there is no propagation, whilst in the fourth frequency range conventional propagation occurs which is indicated by the propagation constant curve positioned on the right side of the light line. In the second and the third frequency ranges, the propagation constant has similar trend, i.e. it exhibits the same behaviour as the genuine SPPs, being positioned on the right side of the light line and going to infinity at specific frequencies called surface-plasmon frequencies *f*_*SP*1_ and *f*_*SP2*_. These frequencies play an important role in filters design since they are spectral positions at which *β* goes to the infinity, which causes propagation suppression and a sharp transmission zero in the structure’s response. We note the region marked with lines in the second range, in which the effective dielectric constant of the middle layer is still negative, however its absolute value is smaller than that of the top layer. Thus, the propagation constant does not go to infinity nor SPP-like nor conventional propagation occurs, i.e. the region represents a stopband range. The same explanation can be applied to the region marked with lines in the third frequency range.

In other words, once an SPP-like propagation is established at the interface between the top and middle layers, propagation exists in the second frequency range, which is interrupted at *f*_*SP1*_ and forbidden in the region marked with lines. Propagation is established again once an SPP-like occurs at the interface between the bottom and middle layers, which is interrupted at *f*_*SP2*_ and forbidden in the corresponding region marked with lines. These transmission zeros together with the described stopband regions are capable to clearly separate passbands in the spectrum, and this is the main principle of the filters’ design.

**Figure 3:**
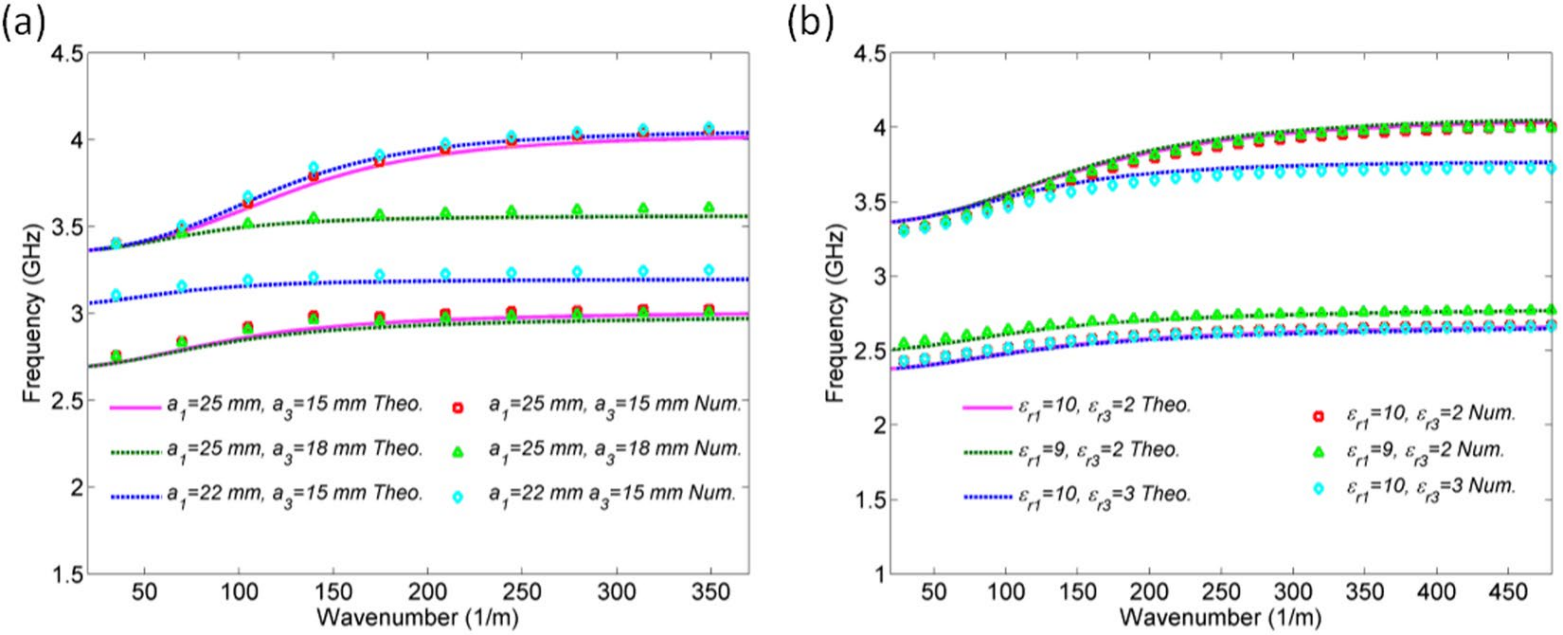
(**a**) Theoretically and numerically obtained dispersion relations for different values of *a*_1_ and *a*_3_. (**b**) Theoretically and numerically obtained dispersion relations for different values of *ε*_*r*1_ and *ε*_*r3*_.

The surface-plasmon frequencies *f*_*SP1*_ and *f*_*SP2*_, spectral width of the ranges in which propagation does not occur and consequently the bandwidths of the passbands, can be controlled by the *sub-SIWs* parameters – dielectric constant and the width. Figure 3(a) shows the theoretically obtained dispersion relations for different values of *a*_*1*_ and *a*_3_, when *ε*_*r1*_ = *ε*_*r*2_ = *ε*_*r3*_ = 5 and *a*_*2*_ = 20 mm, whilst Fig. 3(b) shows the theoretically obtained dispersion relations for different values of *ε*_*r*1_ and *ε*_*r3*_, when *ε*_*r*2_ = 5 and *a*_*1*_ = *a*_*2*_ = *a*_3_ = 20 mm. Other geometrical parameters are kept constant. The parameter *a*_1_ affects only *f*_*SP1*_ and the region marked with lines in the second frequency range, i.e. the first passband, whilst *a*_3_ affects only *f*_*SP2*_ and the region marked with lines in the third frequency range, i.e. the second passband. Similarly, *ε*_*r*1_ and *ε*_*r3*_ affect only the first and the second passband, respectively. Therefore, the widths and dielectric constants of *sub-SIWs* represent mechanisms to independently control the passbands’ positions and bandwidths, which is of crucial importance in dual-band filter design.

### Filter design

To demonstrate the potential of the SPP-like propagation and the three-layer SIW structure for dual-band filtering operation, two dual-band filters have been designed. Since the *sub-SIWs’* width and dielectric constants are mechanisms for independent control of the passbands, theoretically there are six degrees of freedom in filter design. However, it should be noted that the top *sub-SIW* should have the lowest cut-off frequency and the bottom one the highest cut-off frequency or vice versa, to achieve dual-band operation. Otherwise, the two SPP-like propagation would overlap and only one passband would be formed. Also, to further simplify the procedure and simultaneously keep the design freedom it is judicious to use either the same width or the same dielectric constant for the three *sub-SIWs*.

Therefore, the first filter that we propose comprises three *sub-SIWs* of the same width filled with different dielectric materials. The dielectric constant of the middle layer and its width have been chosen to position its cut-off frequency in the range 2.7–3.2 GHz to be able to position the passbands around 2.4 and 3.5 GHz. We note that these frequencies correspond to the IEEE standards, but in general they are arbitrarily chosen. Afterwards, the dielectric constants of the top and bottom *sub-SIWs* have been chosen to obtain the passbands at the desired frequencies. Since the three layers have different dielectric materials they are realized using the following dielectric substrates: Rogers TMM10i with relative permittivity *ε*_*r*1_ = 9.8, dielectric loss *tanδ*_*1*_ = 0.002, and thickness *t*_*1*_ = *b-d-τ* = 1.27 mm, Neltec NH9450 with relative permittivity *ε*_*r*2_ = 4.5, dielectric loss *tanδ*_*2*_ = 0.0027, and thickness *τ* = 0.768 mm, and Rogers RT5880 with relative permittivity *ε*_*r*_ = 2.2, dielectric loss *tanδ* = 0.0009, and thickness *d* = 0.51 mm. Also, it should be noted that using of the commercially available substrates somewhat limit the range of the available dielectric constants, and consequently the design freedom. However, this does not affect the overall idea and this limitation can be overcome by variation of the widths.

After the three *sub-SIWs* are designed based on theoretical analysis, numerical simulations are used to finely tune the structure’s response. Although the theory can very precisely determine the positions and bandwidths of the passbands, the filter needs to be numerically optimized since the theoretical model cannot predict minute mutual couplings between the layers that affect the filter response. Also, good impedance matching in the whole range of interest is very important for filter performance and thus the microstrip-to-SIW transition has to be optimized.

We note here that the *sub-SIWs* are mutually coupled through arrays of wires as indicated in Fig. 1, since the wires provide that there are no other modes which would interfere with TE_10_ mode and disturb the field distribution at the interfaces. Also, the wires support accumulation of electric charges and consequently provide that the normal components of the electric field at the interfaces are opposite to each other. If there were not wires, then the whole structure would behave as a conventional partially filled waveguide which cannot provide SPP propagation but only conventional propagation. In other words, the interface itself is not sufficient to achieve the coupling between two TE_10_ modes, but the SPP-like propagation has to be reinforced through the array of wires.

The final geometrical parameters of the filter are the following: *a* = 22 mm, *L* = 40 mm, *L*_*T*_ = 12.5 mm, *W*_*in*_ = 4.5 mm, *W*_*t*_ = 5 mm, *w*_*w*_ = 0.2 mm, *d*_*w*1_ = 1 mm, *n*_*w1*_ = 33, *d*_*w2*_ = 0.4 mm, *n*_*w2*_ = 65, *d*_*via*_ = 0.8 mm, *p*_*via*_ = 1.1 mm, where *d*_*via*_ represents the diameter of the via, *p*_*via*_ the spacing between the vias, whilst *n*_*w1*_ *and n*_*w2*_ represent the number of wires at the interface between neighbouring materials.

Since the actual configuration of the filters employs the arrays of wires, which were not considered in the theoretical analysis, at this point we would like to show that the introduction of the wires, instead of ideal SPP interfaces, does not significantly influence the SPP-like propagation. To that end, Fig. 3 also shows the comparison of the theoretically predicted dispersion relations with those in which the wires are taken into account. The latter dispersion relations have been obtained using numerical analysis, which is explained in the section Methods, and the geometrical parameters of the wires are those given for the final filter. One can note an excellent agreement between the theoretically and numerically obtained dispersion relations, which confirms that the introduction of wires does not influence the control mechanisms for the filter response.

**Figure 4:**
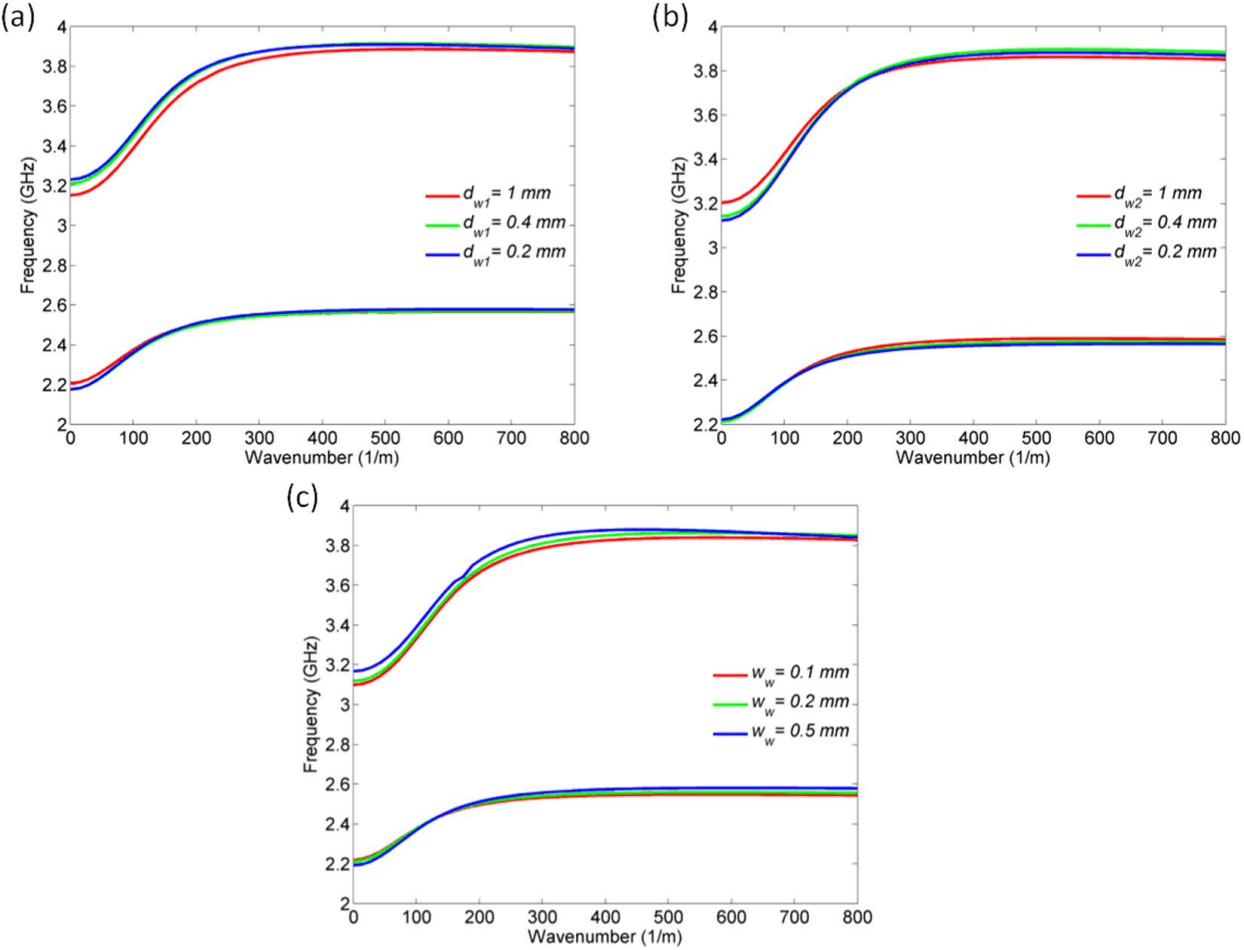
Numerically obtained dispersion relations for different values of the parameter: (**a**) *d*_*w1*_. (**b**) *d*_*w*2_. (**c**) *w*_*w*_.

However, one may argue that different periodicity of the wires and their geometrical parameters can influence the overall structure behaviour. To avoid any doubt, Fig. 4 shows numerically obtained dispersion relations for different values of the parameters *d*_*w1*_, *d*_*w2*,_ and *w*_*w*_. The dispersion relations exhibit very similar trend and values, particularly in the region in which *β* goes to the infinity, which means that the geometrical parameters practically do not influence the positions of the transmission zeros induced by the SPP-like propagation. Certainly, the previous claim is valid as far as the periodicity of the wires is significantly smaller than the operational wavelength.

Nevertheless, the geometrical parameters of the wires at the two interfaces differ, since they have been optimized to achieve minimal loss in the filter response, i.e. to achieve the sufficiently good impedance matching.

**Figure 5:**
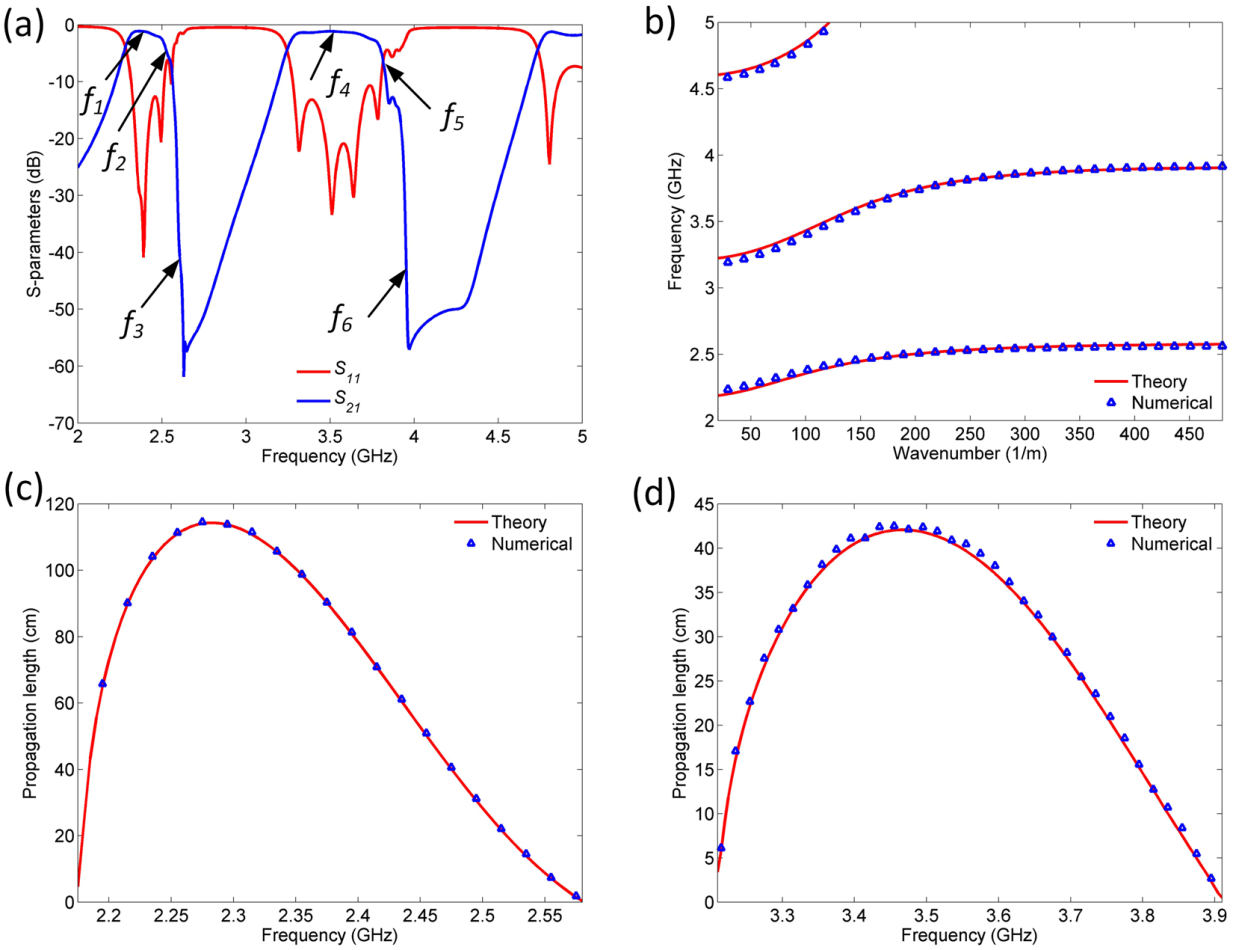
(**a**) Simulation response of first proposed filter. (**b**) Theoretically and numerically obtained dispersion relations of the first proposed filter. **(c)** Theoretically and numerically calculated propagation length of the first SPP-like mode. **(d)** Theoretically and numerically calculated propagation length of the second SPP-like mode.

Figure 5(a) shows the response of the filter. The central frequencies of the two passbands are positioned at 2.4 and 3.5 GHz, and their 3-dB bandwidths are 10.3% and 15.8%, respectively. The insertion losses are 1.1 and 1.2 dB, whereas the return losses are below 13 and 11 dB, respectively. The passbands are characterized by the excellent selectivity which is primarily due to transmission zeros that occur at surface-plasmon frequencies.

Theoretically and numerically obtained dispersion diagrams of the structure are shown in Fig. 5(b). The theoretically obtained dispersion diagram confirms the validity of the theoretical model since the passbands, stopbands, and transmission zeros in the response excellently agree with the model. This is further supported by the numerically obtained dispersion diagram, which shows a very good agreement with the theoretical one.

In addition, Fig. 5 shows the analytically and numerically obtained propagation length of the SPP-like modes around the central frequencies of the two passbands, both of which have been calculated using the expression^38^:

5 $$L=Q\frac{{v}_{g}}{Re(\omega )}=\frac{1}{2Im(\beta )}.$$

The parameter *v*_*g*_ is the group velocity obtained as derivative of the dispersion relation, *ω* is the complex eigenfrequency, *β* propagation constant, and *Q* is the quality factor of SPP-like mode calculated as *Q* = *Re(ω)/2Im(ω)*. Theoretical and numerical results show an excellent agreement. Also, both SPP-like modes exhibit relatively long propagation lengths, with the greatest values equal to 117.1 and 43.2 cm, respectively.

**Figure 6:**
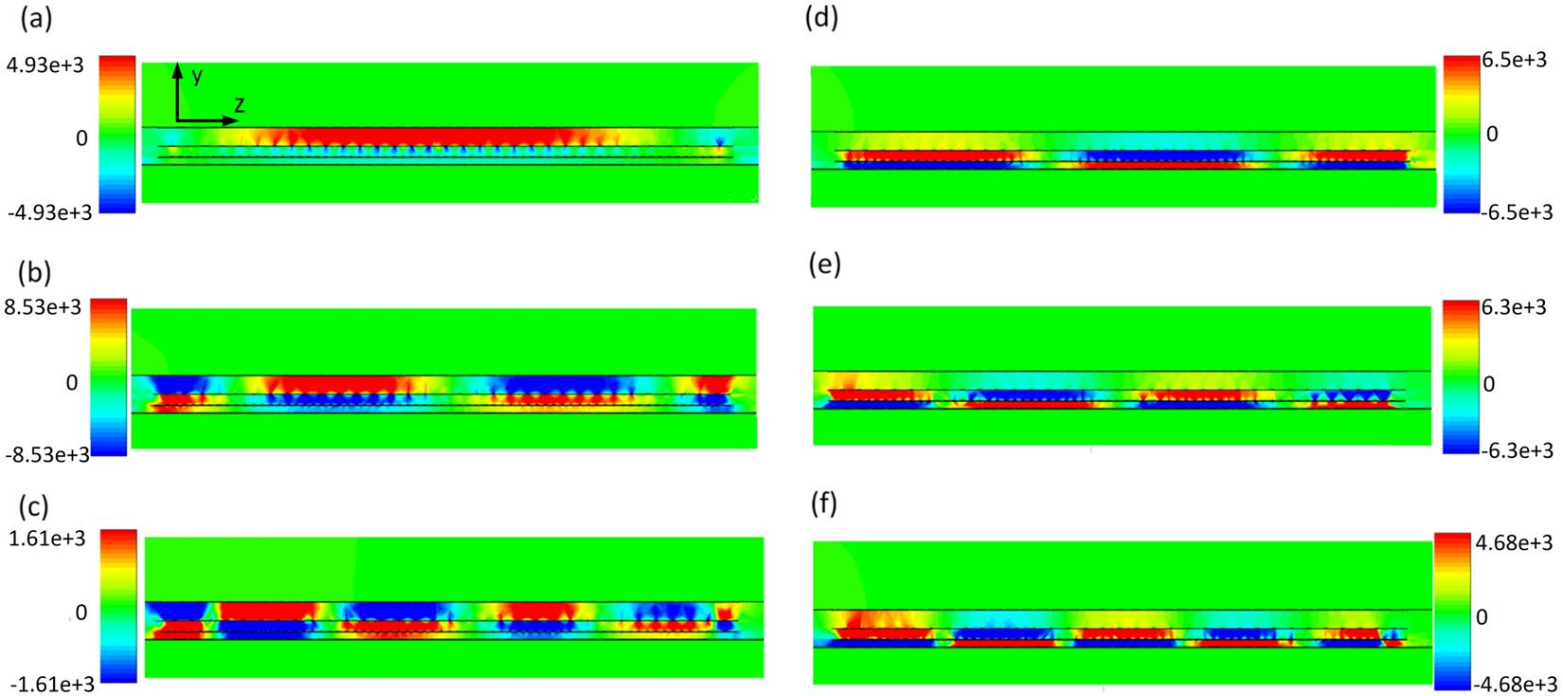
Electric field distribution in first proposed filter at *y-z* cross section along the line of symmetry at: (**a**) *f*_*1*_. (**b**) *f*_*2*_. (**c**) *f*_3_. (**d**) *f*_4_. (**e**) *f*_5_. (**f**) *f*_6_. The unit of the shown electric field is V/m.

The SPP-like behaviour is also confirmed in Fig. 6 that shows the electric field distribution at the interfaces at the frequencies denoted in Fig. 5. Whilst E-field distribution at *f*_*1*_ and *f*_4_ is very similar to that of the conventional SIW, at *f*_2_, *f*_*3*,_ *f*_5_, and *f*_6_ the wavelength is significantly smaller implying increase of *β* and its approaching to the infinity. At *f*_1_ and *f*_4_ the absolute values of dielectric constants in the corresponding layers are still sufficiently different, whilst at *f*_2_ and *f*_5_, and particularly at *f*_3_ and *f*_6_ they have practically the same absolute values causing *β* to abruptly increase.

The second filter that we propose comprises three *sub-SIWs* with different widths and same dielectric constants. To that end, the dielectric substrate Neltec NH9450 with relative permittivity *ε*_*r*2_ = 4.5, dielectric loss *tanδ*_*2*_ = 0.0027, and thickness *τ* = 0.768 mm, has been used. The widths *a*_1_, *a*_2_, and *a*_3_ have been determined to achieve the passbands around the frequencies 4.7 and 5.5 GHz. Following the theoretical model, numerical analysis and simulations have been performed to optimize the filter response in terms of impedance matching and suppressing non-desirable coupling. To that end, the microstrip-to-SIW transmission has been optimized and strips between the arrays of vias in different layers added. As in the previous case, the *sub-SIWs* are mutually coupled through the array of wires to achieve the SPP-like propagation. The final geometrical parameters of the filter are the following: *a*_1_ = 16 mm, *a*_2_ = 13.8 mm, *a*_3_ = 12.4 mm, *L* = 25 mm, *L*_*T*_ = 5 mm, *W*_*in*_ = 4.5 mm, *W*_*t*_ = 9 mm, *w*_*w*_ = 0.25 mm, *d*_*w*1_ = 0.25 mm, *n*_*w1*_ = 45, *d*_*w*2_ = 0.375 mm, *n*_*w2*_ = 30, *d*_*via*_ = 0.5 mm, *p*_*via*_ = 0.8 mm.

**Figure 7:**
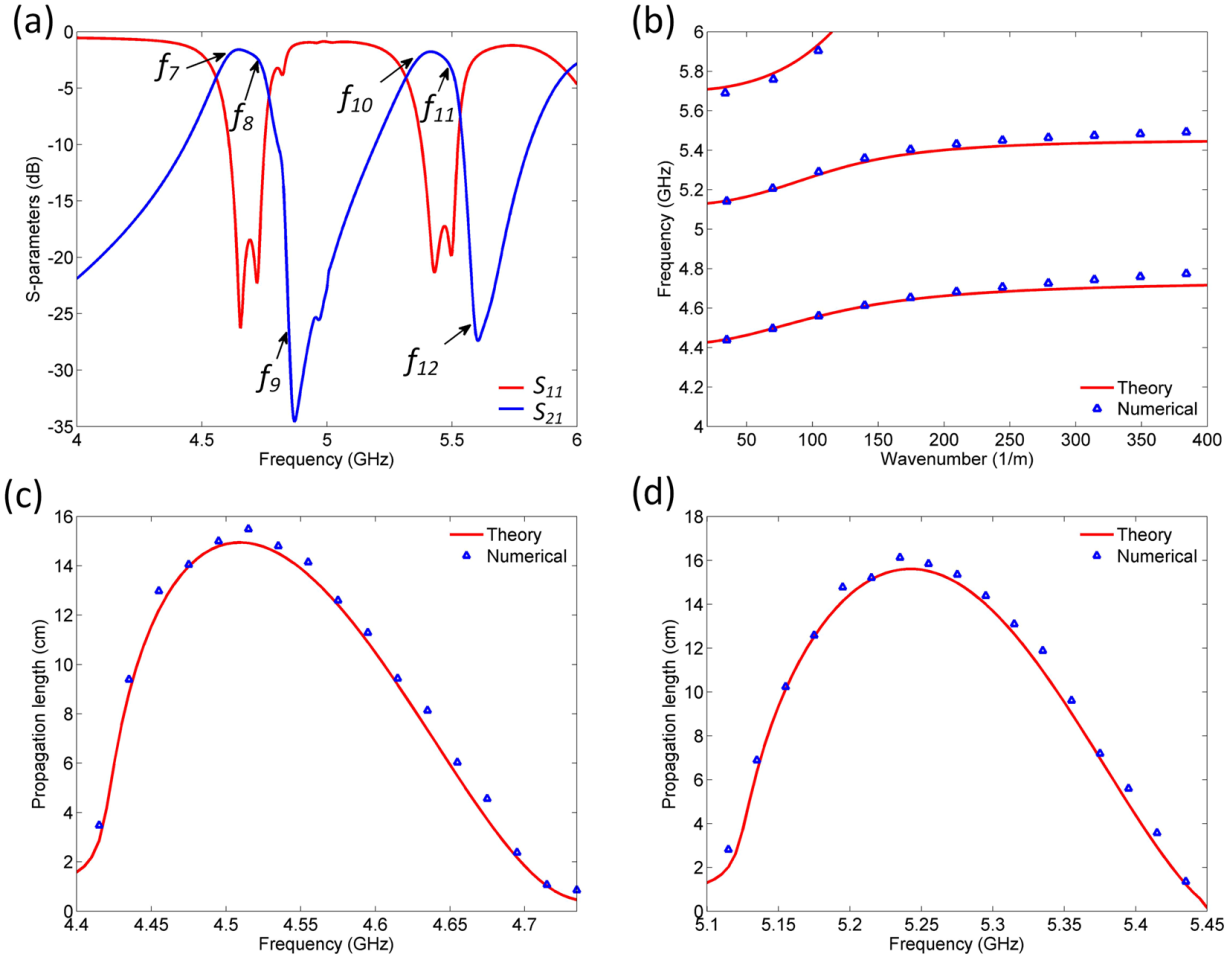
(**a**) Simulation response of second proposed filter. (**b**) Theoretically and numerically obtained dispersion relations of the second proposed filter. **(c)** Theoretically and numerically calculated propagation length of the first SPP-like mode. **(d)** Theoretically and numerically calculated propagation length of the second SPP-like mode.

**Figure 8:**
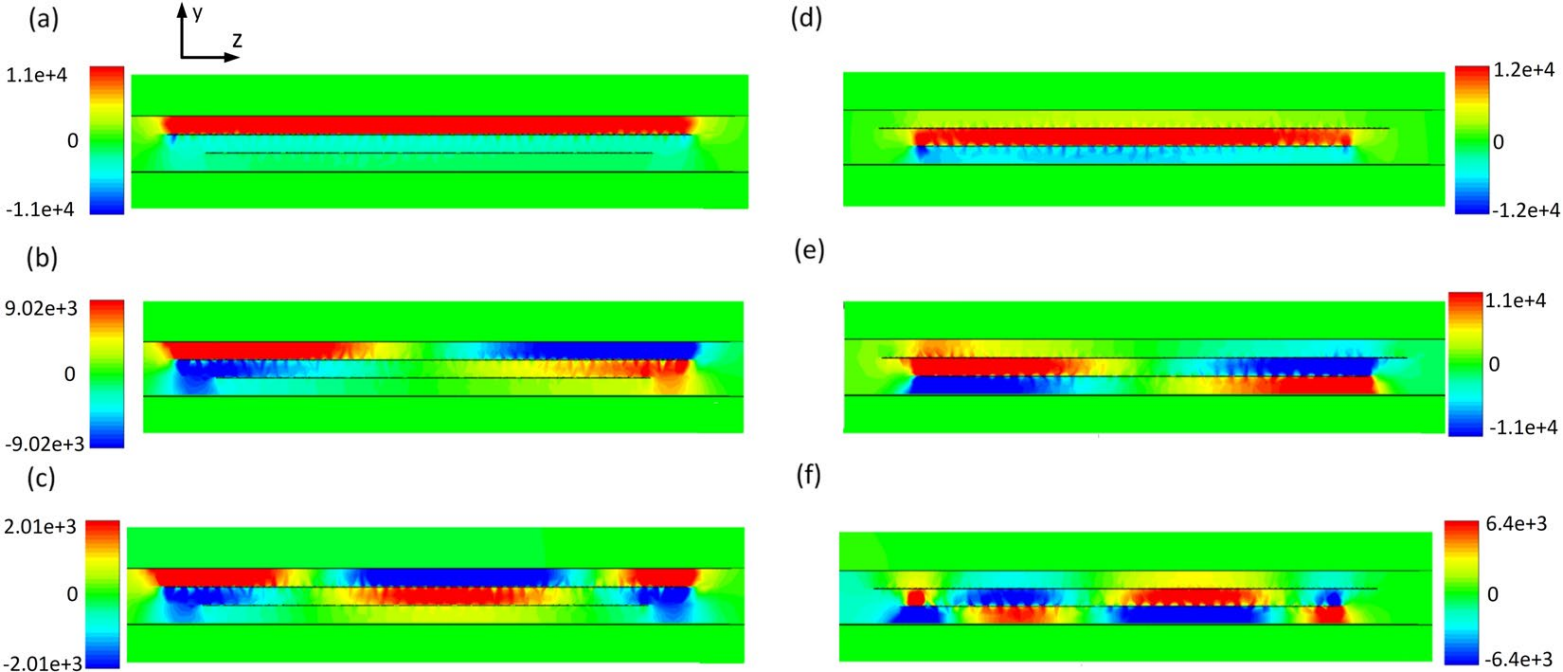
Electric field distribution in second proposed filter at *y-z* cross section along the line of symmetry at: (**a**) *f*_7_. (**b**) *f*_8_. (**c**) *f*_9_. (**d**) *f*_10_. (**e**) *f*_11_. (**f**) *f*_12_. The unit of the shown electric field is V/m.

Figure 7 shows the response of the filter together with the theoretically and numerically obtained dispersion diagram of the structure and propagation lengths of the SPP-like modes. The central frequencies of the two passbands are positioned at 4.7 and 5.5 GHz, and the 3-dB bandwidths are 4.5% and 4.1%, respectively. The insertion losses are equal to 1.58 and 1.77 dB, whereas the return losses are below 18 and 17 dB, respectively. The passbands are also characterized by the excellent selectivity. The theoretical dispersion diagram confirms the validity of the theoretical model, which is further confirmed by the numerical dispersion diagram. Besides an excellent agreement between the theoretically and numerically obtained propagation lengths, one can note that the propagation lengths of SPP-like modes are somewhat shorter than those in the previous filter, which is due to the higher dielectric losses in the second filter. Figure 8 shows the electric field distribution at the interfaces at the frequencies denoted in Fig. 7, which clearly confirms SPP-like behaviour of the proposed structure.

### Measurement results and discussion

**Figure 9:**
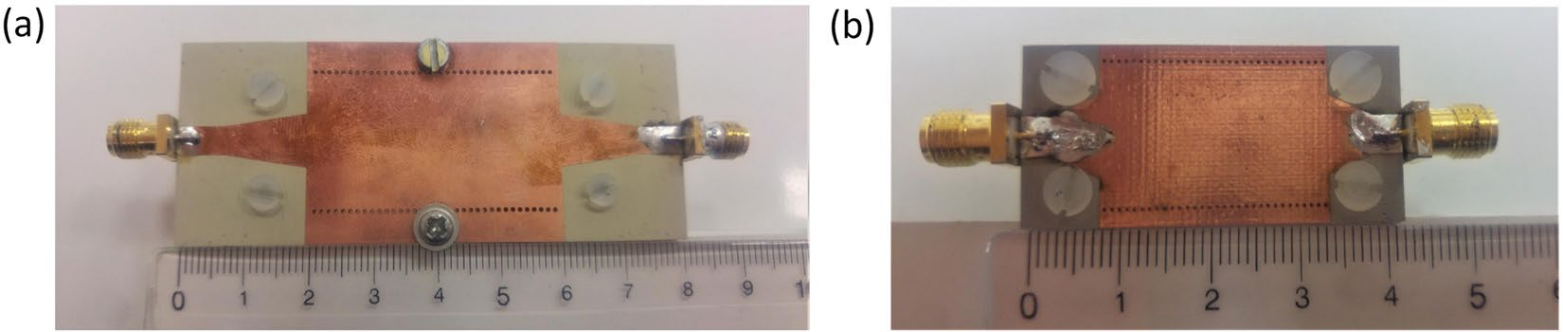
Fabricated filters. (**a**) First proposed filter. (**b**) Second proposed filter.

To validate the designed filters, they have been fabricated using standard printed circuit board (PCB) technology, which is explained in detail in the section Methods. Photographs of the fabricated circuits are shown in Fig. 9, whilst the photographs of the individual layers of the filters are shown in Supplementary Material.

As it is explained in the section Methods, the three layers of each filter are fabricated separately and afterwards assembled into the final structure using screws, which causes small air gaps between the layers. Since SPP-like propagation is confined to the interface between the layers, it is very sensitive to the existence of small disturbances around the interface. Therefore, the air gaps, although very thin, can influence the spectral positions of the passbands. Although their thickness cannot be precisely determined, the air gaps have been approximated by the thickness of the copper layer, i.e. 0.035 mm, which might be taken as the limit by which the layers can be screwed.

**Figure 10:**
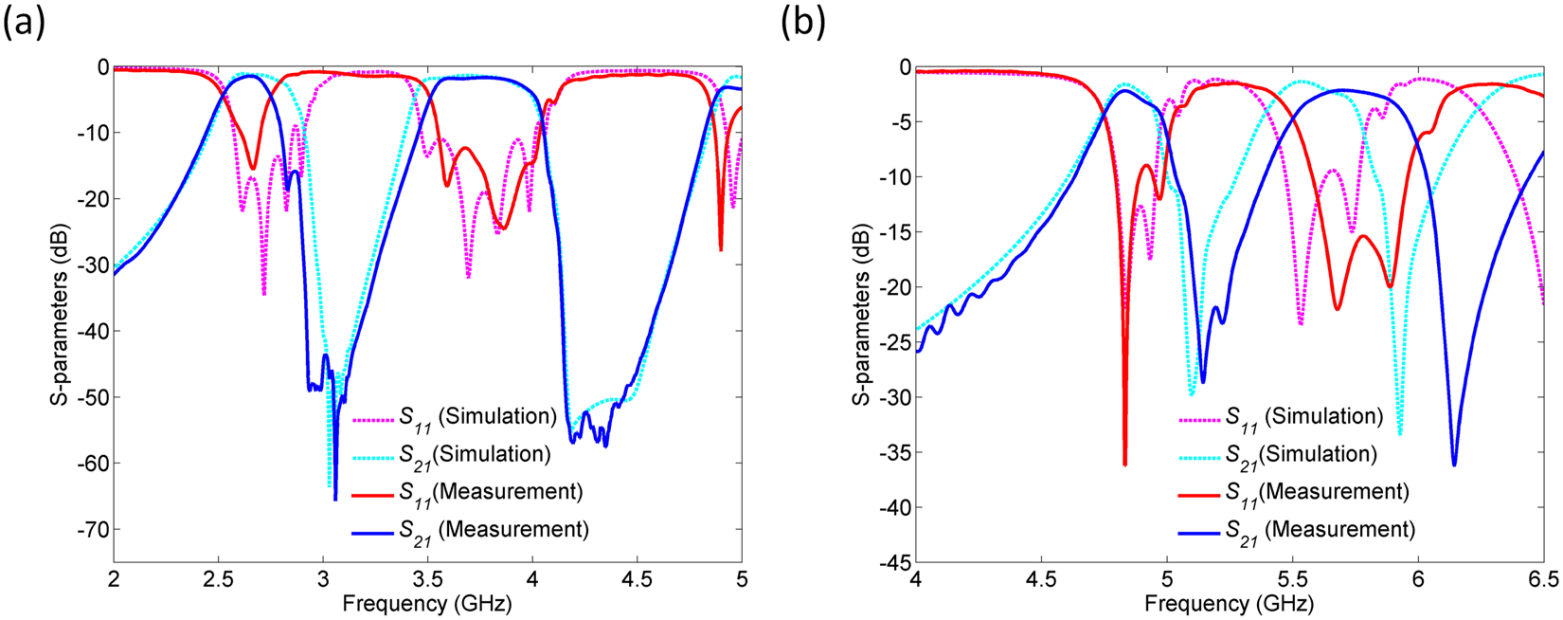
Comparison of the measurement and simulation results: (**a**) First proposed filter. (**b**) Second proposed filter.

In accordance with the previous approximation, Fig. 10 shows the simulation results that correspond to the designed structures in which the air gaps between the layers are taken into account. If these simulation results are compared to those in Figs 5 and 7, the same trend of the curves can be noted, except for the spectral shifts of around 0.2 GHz. The comparison of the measurement and simulation results in Fig. 10 reveals a good agreement except for slight differences in the bandwidths which can be attributed to the tolerance of the manufacturers related to the dielectric constant, as well as to a slight misalignment of the via arrays in fabrication process.

The first filter exhibit two passbands at 2.65 and 3.75 GHz, with insertion loss of 1.47 and 1.69 dB, and the 3-dB bandwidths of 8.7% and 13.3%, respectively. The central frequencies of the second filter are 4.8 and 5.7 GHz, their insertion losses 2.22 and 2.17 dB, and the 3-dB bandwidths 5.2% and 8.2%, respectively. Both filters are characterized by good in-band and out-of-band performance as well as by excellent selectivity owing to the transmission zeros. The difference in the insertion losses between the first and the second filter can be attributed to the fact that the first filter comprises three different substrates whose dielectric losses are *tanδ*_*1*_ = 0.002, *tanδ*_*2*_ = 0.0027, and *tanδ*_3_ = 0.0009, respectively, whilst in the second proposed filter only the substrate with *tanδ*_2_ = 0.0027 has been used. Therefore, the losses in dielectric are more pronounced in the second proposed filter, which results in higher insertion losses.

The proposed filters are first dual-band filters based on the “natural” SPP-like concept, and they are characterized by good overall performance and possibility for independent control of the passbands in terms of position and bandwidth. Moreover, they meet requirements for low-cost and low-profile configuration since they are realized as SIW structures. As such, they can be considered as very promising candidates for dual-band filtering operation at microwave frequencies.

## Conclusion

In this work, we proposed novel microwave dual-band filters employing SPP-like propagation based on structural dispersion of SIW. The filters are realized as a SIW that consists of three *sub-SIW* structures, tailored to exhibit effective permittivities of opposite signs in certain frequency ranges. Such configuration provides two distinct SPP-like propagations to occur, ultimately providing two passbands in the filters’ responses.

A detailed theoretical and numerical analysis of the structures’ behaviour have been presented and they showed how the choice of the geometrical parameters and dielectric constants of *sub-SIWs* can provide arbitrarily positioning of the passbands in the spectrum.

To demonstrate the potential of the proposed ideas, we have designed two dual-band filters operating at 2.65/3.75 and 4.8/5.7 GHz, respectively, which are characterized by excellent in-band characteristics and selectivity. The designed filters have been fabricated using standard PCB technology, and the measurements results agree well with the simulated ones.

The proposed filters are first dual-band microwave filters based on SPP-like propagation and they are excellent candidates for dual-band filtering applications since they provide independent control of the passbands, excellent performance, and low-cost fabrication.

## Methods

### Design optimization

The responses of the structures were simulated and optimized using FEM method employed in the commercial software package CST. Both structures were simulated using frequency domain solver and tetrahedral mesh in the frequency ranges from 2 to 5 GHz and 4 to 6 GHz, respectively.

To properly configure the feeding of the filters and achieve appropriate impedance matching, in the initial step we determined the optimal widths of the microstrip lines *W*_*in*_ and SIW feeding lines *W*_*t*_ using quasi-static expressions^39–41^. Since there is a difference between the widths *W*_*in*_ and *W*_*t*_, a microstrip taper is used and configured to have a smooth transition from one width to another. Due to the complexity of the structure and the dual-band operation, in the final step the analytically designed feeding scheme was numerically optimized to achieve a good impedance matching in both passbands.

Dispersion curves have been numerically obtained using unit cell configuration and eigenmode solver in which periodic boundary conditions were used in the direction of propagation. Unit cells comprise the three dielectric layers and a wire for each interface between the layers. The eigenmode analysis was conducted using both CST and COMSOL Multiphysics solvers.

### Fabrication

The first structure has been fabricated using standard printed circuit board (PCB) technology. The three layers have been realized using the commercially available substrates Rogers TMM10i with relative permittivity *ε*_*r*1_ = 9.8, dielectric loss *tanδ*_1_ = 0.002, and thickness *t*_*1*_ = *b – d – τ* = 1.27 mm, Neltec NH9450 with relative permittivity *ε*_*r*2_ = 4.5, dielectric loss *tanδ*_*2*_ = 0.0027, and thickness *τ* = 0.762 mm, and Rogers RT5880 with relative permittivity *ε*_*r*3_ = 2.2, dielectric loss *tanδ*_*3*_ = 0.0009, and thickness *b* = 0.51 mm. Metallic parts of each layer were etched according to the design and the three layers have been assembled using six nylon screws. In the final step, SMA connectors have been mounted on the structure.

The second filter has been fabricated using the same technology. Three layers of the substrate Neltec NH9450 with relative permittivity *ε*_*r*2_ = 4.5, dielectric loss *tanδ*_*2*_ = 0.0027 have been used to realize the filter. Metallic parts of each layer were etched according to the design and the three layers have been assembled using four nylon screws. In the final step, SMA connectors have been mounted on the structure.

### Measurements

The responses of the fabricated structures were measured using Vector Network Analyzer E5071C two-port measurements. Measurements were performed with the resolution of 1601 points in the frequency range from 2 to 5 GHz and from 4 to 6.5 GHz, respectively.

## Electronic supplementary material

Supplementary info
