# Supplementary material for: Novel Dual-band Band-Pass Filters Based on Surface Plasmon Polariton-like Propagation Induced by Structural Dispersion of Substrate Integrated Waveguide
Source: Sci Rep. 2018 May 29;8:8332. doi: 10.1038/s41598-018-26705-w (PMC5974233; doi:10.1038/s41598-018-26705-w)
Supplement: Supplementary file 1 — Supplementary info [file 41598_2018_26705_MOESM1_ESM.pdf]

Novel Dual-band Band-Pass Filters Based on Surface Plasmon Polariton-like Propagation Induced by Structural Dispersion of Substrate Integrated Waveguide

Norbert Cselyuszka*, Zarko Sakotic, Goran Kitic, Vesna Crnojevic-Bengin, Nikolina Jankovic

BioSense Institute—Research Institute for Information Technologies in Biosystems, University of Novi Sad, Dr Zorana Djindjica 1a, 21101 Novi Sad, Serbia

* Corresponding Author: cselyu@biosense.rs

Supplementary Material

Derivation of Equation (3) in the manuscript

As illustrated in Figure S1, the proposed structure is a substrate integrated waveguide (SIW) which is comprised of three different layers (1, 2, 3) filled with nonmagnetic, isotropic, and homogeneous dielectric materials with relative permittivity ε_{r1}, ε_{r2}, and ε_{r3}, respectively.

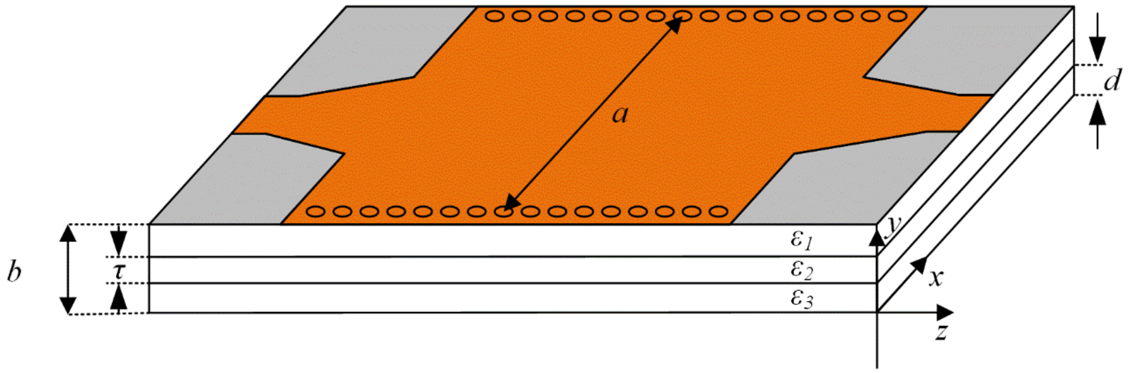

Figure S1: Layout of the proposed filters.

The substrate integrated waveguide supports the transverse electric (TE) modes propagating along the *z* direction and they are characterized by effective dielectric constant due to structural dispersion of SIW. The effective dielectric constant of the dominant TE_{10} mode in each layer can be defined as:

$$\varepsilon_{ei} = \varepsilon_{ri} - \left(\frac{c}{2af}\right)^2 \qquad \text{(S1)}$$

where *f* is the frequency, *a* is the width of the waveguide, *c* is the speed of light, and ε_{ri} and ε_{ei} are relative and effective dielectric constants of each layer (*i* = 1, 2, 3), respectively. To derive the dispersion relation, we set the electric field component along the *z* direction in each layer as[1-2]

$$E_z = \left(A_+ e^{k_{y3} y} + A_- e^{-k_{y3} y}\right) e^{-j\beta z} \qquad \text{for } 0 < y < d$$

$$E_z = \left(B_+ e^{k_{y2}\left(y-d-\frac{\tau}{2}\right)} + B_- e^{-k_{y2}\left(y-d-\frac{\tau}{2}\right)}\right) e^{-j\beta z} \qquad \text{for } d < y < d+\tau \qquad \text{(S2)}$$

$$E_z = \left(C_+ e^{k_{y1}(b-y)} + C_- e^{-k_{y1}(b-y)}\right) e^{-j\beta z} \qquad \text{for } d+\tau < y < b$$

where A_+, A_-, B_+, B_-, C_+, and C_- are the amplitudes of the decaying fields, and *d*, *τ*, and *b* are the geometrical parameters of the structure as shown in Figure S1, *β* is the propagation constant along *z* direction and k_{iy} is the wave-vector component along *y* direction in each layer (*i* = 1, 2, 3) defined as:

$$k_{yi} = \sqrt{\beta^2 - k_0^2 \varepsilon_{ei}} \qquad \text{(S3)}$$

where k_0 is the wave-vector in vacuum. The corresponding electric field in *y* direction E_y and magnetic field H_x can be calculated from the electric field E_z and written as:

$$E_y = j\frac{\beta}{k_{y3}}\left(A_+e^{k_{y3}y} - A_-e^{-k_{y3}y}\right)e^{-j\beta z} \qquad \text{for } 0 < y < d$$

$$E_y = j\frac{\beta}{k_{y2}}\left[B_+e^{k_{y2}\left(y-d-\frac{\tau}{2}\right)} - B_-e^{-k_{y2}\left(y-d-\frac{\tau}{2}\right)}\right]e^{-j\beta z} \qquad \text{for } d < y < d+\tau \qquad \text{(S4)}$$

$$E_y = j\frac{\beta}{k_{y1}}\left(C_-e^{-k_{y1}(b-y)} - C_+e^{k_{y1}(b-y)}\right)e^{-j\beta z} \qquad \text{for } d+\tau < y < b$$

$$H_x = j\frac{\omega\varepsilon_0\varepsilon_{e3}}{k_{y3}}\left(A_+e^{k_{y3}y} - A_-e^{-k_{y3}y}\right)e^{-j\beta z} \qquad \text{for } 0 < y < d$$

$$H_x = j\frac{\omega\varepsilon_0\varepsilon_{e2}}{k_{y2}}\left[B_+e^{k_{y2}\left(y-d-\frac{\tau}{2}\right)} - B_-e^{-k_{y2}\left(y-d-\frac{\tau}{2}\right)}\right]e^{-j\beta z} \qquad \text{for } d < y < d+\tau \qquad \text{(S5)}$$

$$H_x = j\frac{\omega\varepsilon_0\varepsilon_{e1}}{k_{y1}}\left(C_-e^{-k_{y1}(b-y)} - C_+e^{k_{y1}(b-y)}\right)e^{-j\beta z} \qquad \text{for } d+\tau < y < b$$

where ε_0 is the dielectric constant of the vacuum. Due to boundary conditions, the tangential component of the electric field E_z has to be zero on metallic walls of the waveguide (y = 0 and $y = b$). Using hyperbolic function identities, the electric field in the layers 1 and 3 can be rewritten as:

$$E_z = Asinh\left(k_{y3}y\right)e^{-j\beta z} \qquad \text{for } 0 < y < d$$

$$E_z = Csinh\left[k_{y1}(b-y)\right]e^{-j\beta z} \qquad \text{for } d+\tau < y < b \qquad \text{(S6)}$$

where due to boundary conditions $A = 2A_+ = -2A_-$ and $C = 2C_+ = -2C_-$. Using the same identities in the expression (S5), the magnetic field H_x in the layers 1 and 3 can be expressed as:

$$H_x = j\frac{\omega\varepsilon_0\varepsilon_{e3}}{k_{y3}}Acosh\left(k_{y3}y\right)e^{-j\beta z} \qquad \text{for } 0 < y < d$$

$$H_x = -j\frac{\omega\varepsilon_0\varepsilon_{e1}}{k_{y1}}Ccosh\left[k_{y1}(b-y)\right]e^{-j\beta z} \qquad \text{for } d+\tau < y < b \qquad \text{(S7)}$$

According to the boundary conditions at the interfaces of different regions ($y = d$ and $y = d+\tau$), where the tangential electric field component E_z and the tangential magnetic field component H_x are continuous, the following system of equations has to be satisfied:

$$E_z(d) = Asinh\left(k_{y3}d\right)e^{-j\beta z} = \left(B_+e^{-k_{y2}\frac{\tau}{2}} + B_-e^{k_{y2}\frac{\tau}{2}}\right)e^{-j\beta z} \qquad \text{(S8)}$$

$$H_x(d) = j\frac{\omega\varepsilon_0\varepsilon_{e3}}{k_{y3}}Acosh\left(k_{y3}d\right)e^{-j\beta z} = j\frac{\omega\varepsilon_0\varepsilon_{e2}}{k_{y2}}\left[B_+e^{-k_{y2}\frac{\tau}{2}} - B_-e^{k_{y2}\frac{\tau}{2}}\right]e^{-j\beta z} \qquad \text{(S9)}$$

$$E_z(d+\tau) = Csinh\left(k_{y1}[d+\tau]\right)e^{-j\beta z} = \left(B_+e^{k_{y2}\frac{\tau}{2}} + B_-e^{-k_{y2}\frac{\tau}{2}}\right)e^{-j\beta z} \qquad \text{(S10)}$$

$$H_x(d+\tau) = -j\frac{\omega\varepsilon_0\varepsilon_{e1}}{k_{y1}}C cosh\big(k_{y1}[d+\tau]\big)e^{-j\beta z} = j\frac{\omega\varepsilon_0\varepsilon_{e2}}{k_{y2}}\left[B_+e^{k_{y2}\frac{\tau}{2}} - B_-e^{-k_{y2}\frac{\tau}{2}}\right]e^{-j\beta z} \quad \text{(S11)}$$

If the equations (S8) and (S10) are divided by the equations (S9) and (S11), the amplitudes *A* and *C* can be eliminated, and the system of equations reduces to:

$$\frac{k_{y3}}{\varepsilon_{e3}}tanh\big(k_{y3}d\big) = \frac{k_{y2}}{\varepsilon_{e2}}\frac{B_+e^{-k_{y2}\frac{\tau}{2}} + B_-e^{k_{y2}\frac{\tau}{2}}}{B_+e^{-k_{y2}\frac{\tau}{2}} - B_-e^{k_{y2}\frac{\tau}{2}}} \quad \text{(S12)}$$

$$-\frac{k_{y1}}{\varepsilon_{e1}}tanh\Big(k_{y1}(b-d-\tau)\Big) = \frac{k_{y2}}{\varepsilon_{e2}}\frac{B_+e^{k_{y2}\frac{\tau}{2}} + B_-e^{-k_{y2}\frac{\tau}{2}}}{B_+e^{k_{y2}\frac{\tau}{2}} - B_-e^{-k_{y2}\frac{\tau}{2}}} \quad \text{(S13)}$$

Using the identities $e^{\rho} = cosh\,\rho + sinh\,\rho$ and $e^{-\rho} = cosh\,\rho - sinh\,\rho$ the right side of the previous equations can be expressed as:

$$\frac{k_{y3}}{\varepsilon_{e3}}tanh\big(k_{y3}d\big) = -\frac{k_{y2}}{\varepsilon_{e2}}\frac{\alpha\,cosh\Big(k_{y2}\frac{\tau}{2}\Big) + \beta\,sinh\Big(k_{y2}\frac{\tau}{2}\Big)}{\beta\,cosh\Big(k_{y2}\frac{\tau}{2}\Big) + \alpha\,sinh\Big(k_{y2}\frac{\tau}{2}\Big)} \quad \text{(S14)}$$

$$-\frac{k_{y1}}{\varepsilon_{e1}}tanh\Big(k_{y1}(b-d-\tau)\Big) = \frac{k_{y2}}{\varepsilon_{e2}}\frac{\alpha\,cosh\Big(k_{y2}\frac{\tau}{2}\Big) - \beta\,sinh\Big(k_{y2}\frac{\tau}{2}\Big)}{\alpha\,sinh\Big(k_{y2}\frac{\tau}{2}\Big) - \beta\,cosh\Big(k_{y2}\frac{\tau}{2}\Big)} \quad \text{(S15)}$$

where $\alpha = B_+ + B_-$ and $\beta = B_- - B_+$. Since the amplitudes are always non-negatives it implies that $\alpha > \beta$, and consequently the dispersion relation can be reduced to:

$$\frac{k_{y3}}{\varepsilon_{e3}}tanh\big(k_{y3}d\big) = -\frac{k_{y2}}{\varepsilon_{e2}}coth\Big(k_{y2}\frac{\tau}{2} + \psi\Big) \quad \text{(S16)}$$

$$-\frac{k_{y1}}{\varepsilon_{e1}}tanh\Big(k_{y1}(b-d-\tau)\Big) = \frac{k_{y2}}{\varepsilon_{e2}}coth\Big(k_{y2}\frac{\tau}{2} - \psi\Big) \quad \text{(S17)}$$

where Ψ is a parameter[3] expressed as $\psi = atanh(\beta/\alpha)$.

Photographs of the individual layers of the fabricated filters

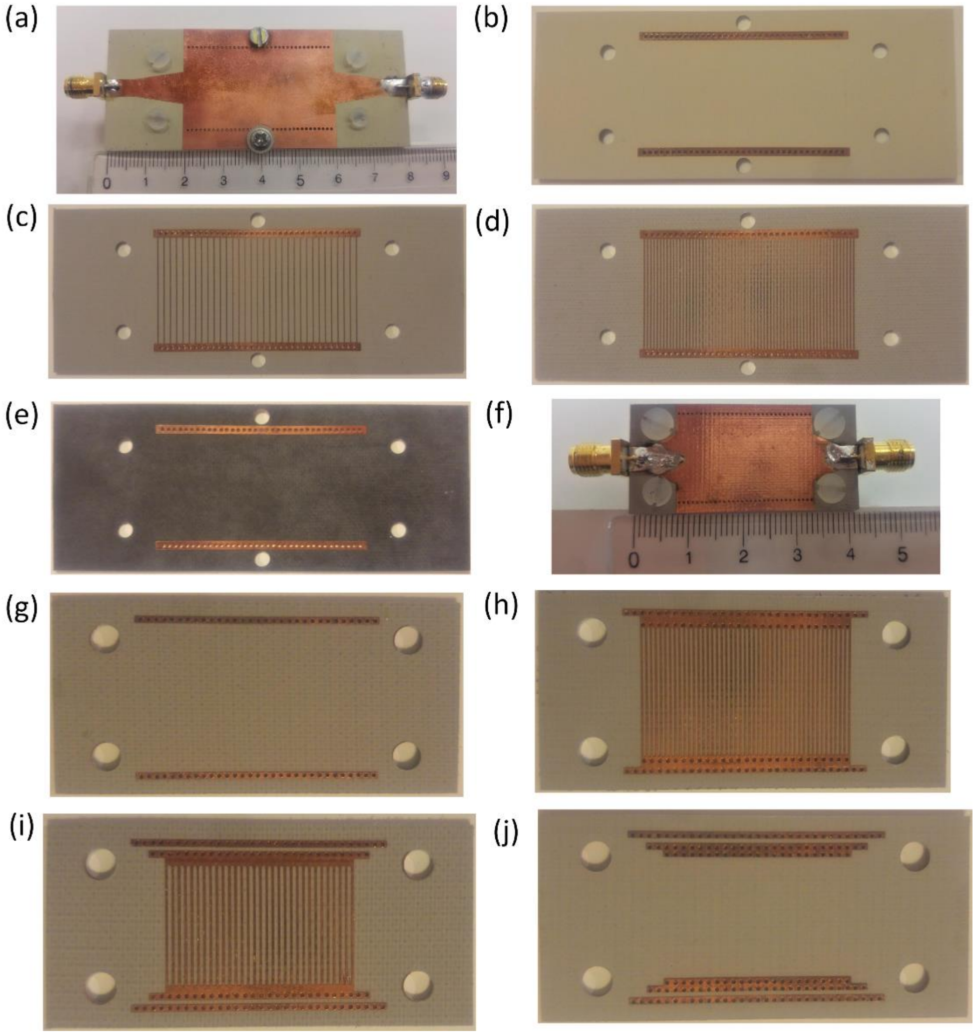

Figure S2. Fabricated filters. (**a**) First proposed filter - top side of the top *sub-SIW*. (**b**) First proposed filter - bottom side of the top *sub-SIW*. (**c**) First proposed filter - top side of the middle *sub-SIW*. (**d**) First proposed filter - bottom side of the middle *sub-SIW*. (**e**) First proposed filter - top side of the bottom *sub-SIW*. (**f**) First proposed filter – overall structure. (**g**) Second proposed filter - top side of the top *sub-SIW*. (**h**) Second proposed filter - bottom side of the top *sub-SIW*. (**i**) Second proposed filter - top side of the middle *sub-SIW*. (**j**) Second proposed filter - bottom side of the middle *sub-SIW*. (**k**) Second proposed filter - top side of the bottom *sub-SIW*. (**l**) Second proposed filter – overall structure.

References:

1. Balanis, C. Advanced engineering electromagnetics, 2nd ed., J. Wiley & Sons, New York (2012).

2. Collin, R. Foundations for microwave engineering, 2nd ed., Wiley-IEEE Press, New York, (2001).

3.Orfanidis, S. J. Electromagnetic Waves and Antennas, available at http://www.ece. rutgers.edu/orfanidi/ewa/
